# Cytip regulates dendritic-cell function in contact hypersensitivity

**DOI:** 10.1002/eji.201041286

**Published:** 2012-01-04

**Authors:** Valeska Heib, Florian Sparber, Christoph H Tripp, Daniela Ortner, Patrizia Stoitzner, Christine Heufler

**Affiliations:** Department of Dermatology and Venereology, Medical University InnsbruckInnsbruck, Austria

**Keywords:** Cellular immunology, Dendritic cells, Hypersensitivity, Immune regulation

## Abstract

Cytohesin-interacting protein (Cytip) is induced during dendritic cell (DC) maturation and in T cells upon activation. It has also been shown to be involved in the regulation of immune responses. Here, we evaluated the functional consequences of Cytip deficiency in DCs using Cytip knockout (KO) mice. No difference in DC subpopulations in the skin draining lymph nodes (LNs) was found between Cytip KO mice and their wild-type counterparts, excluding a role in DC development. To investigate the function of Cytip in DCs in vivo, we used 2,4,6-trinitrochlorobenzene (TNCB)-induced contact hypersensitivity (CHS) as a model system. In the sensitization as well as in the elicitation phase, DCs derived from Cytip KO mice induced an increased inflammatory reaction indicated by more pronounced ear swelling. Furthermore, IL-12 production was increased in Cytip KO bone marrow-derived DCs (BMDCs) after CpG stimulation. Additionally, Cytip-deficient DCs loaded with ovalbumin induced stronger proliferation of antigen-specific CD4^+^ and CD8^+^ T cells in vitro. Finally, migration of skin DCs was not altered after TNCB application due to Cytip deficiency. Taken together, these data suggest a suppressive function for Cytip in mouse DCs in limiting immune responses.

## Introduction

Dendritic cells (DCs) play a major role in regulating immune responses in quality and quantity. As professional antigen-presenting cells with a variety of costimulatory molecules and cytokine production they are predestined to make the choice between tolerance and immunity [1].

Allergic contact dermatitis or contact hypersensitivity (CHS) is one of the most common inflammatory skin diseases induced by repeated contact to low molecular weight chemicals, termed haptens. Understanding the development of CHS and preventing this life-affecting disease is of great importance not only for CHS patients, but would also have a great socioeconomic impact due to, for example, high therapeutic costs. CHS can be divided into two major phases, the sensitization and elicitation phases. During sensitization, skin DCs mature and migrate to the draining lymph nodes (LNs) where they present the hapten to naive T cells, which leads to the development of antigen-specific effector T cells [2]. During elicitation, reexposure to the same hapten leads to the activation of effector T cells. These T cells induce an inflammatory process responsible for cutaneous lesions. Inflammation peaks at 24–48 h and decreases very rapidly. The activation of T cells requires three different signals: presentation of hapten by DCs, costimulatory signals by molecules expressed on DCs, and cytokines secreted during DC–T-cell interactions. To avoid an overreaction of activated T cells, negative signals have also to be delivered during DC–T-cell contact [3, 4].

The cytohesin-interacting protein (Cytip) was shown to play a role in the detachment of human DCs and T cells [5]. Hofer et al. [5] showed that Cytip accumulates at the contact zone between human DCs and T cells, and that activation of CD8^+^ T cells is diminished when Cytip is silenced in DCs in co-cultures with CD8^+^ T cells. They also demonstrated that DCs use Cytip to actively limit the contact to T cells. It was also shown that Cytip regulates lymphocyte function-associated antigen (LFA) –1–ICAM-1-mediated adhesion by binding to cytohesin-1 via the coiled-coil domains of both molecules and the translocation of cytohesin-1 from the membrane to the cytosol [6]. When cytohesin-1 is translocated from the membrane to the cytosol, LFA-1 is inactivated on DCs [6]. Using a Cytip knockout (KO) mouse, Coppola et al. [7] showed that Cytip does not affect the development of the immune system, although they found fewer white blood cells and lymphocytes in the LNs. The same group also demonstrated that the immune response in a virus-induced tumor model (M-MSV) is reduced in Cytip-deficient mice [7]. Another Cytip-deficient mouse, published by Watford et al. [8], also showed no effect in the development of the immune system.

Due to these findings, we wanted to investigate the function of Cytip in mouse DCs and how it might influence the regulation of CHS by DCs.

## Results

### Cytip deficiency does not alter DC development

**Figure 1:**
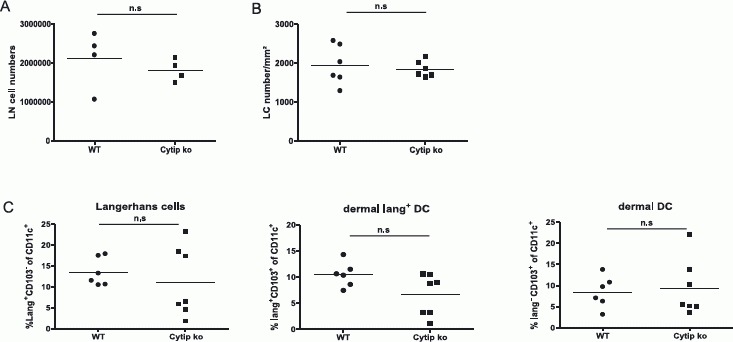
DC subpopulations in skin draining LNss are not affected by Cytip deficiency. Auricular LNs were taken from wild type (WT) and Cytip-deficient mice and cell suspensions were prepared. (A) The viable cell numbers were determined with a haemocytometer. (B) To examine the number of Langerhans cells (LCs) in the epidermis, epidermal sheets were prepared and stained for MHC class II and LCs were counted. (C) Skin draining LN cells were stained for CD11c, CD40, Langerin, and CD103 to investigate the different DC populations LCs (Lang^+^CD103^−^), Langerin^+^ dermal DCs (Lang^+^CD103^+^) and dermal DCs (Lang^−^CD103^+^). Bars represent the mean. One experiment representative of three independent experiments (3–5 mice per experiment) with similar results is shown.

As DCs are the major cell type in initiating CHS, we investigated the functional role of Cytip in DCs in more detail. Therefore, cell suspensions from skin draining LNs were prepared and living cells were counted. As depicted in Fig. 1, absolute cell number in skin draining LNs was not altered in Cytip KO mice compared with that of their wild-type (WT) counterparts (Fig. 1A). Because of publications with opposite results regarding the respective role of Langer-hans cells (LCs) in skin immunity and especially in CHS [9–11], LC numbers in the epidermis were assessed by staining epidermal sheets for major histocompatibility complex (MHC) class II and counting-positive cells (Fig. 1B). Both WT and Cytip KO show a similar number of LCs in the epidermis.

DCs in skin draining LNs can be subdivided into three major populations; LCs, langerin-positive dermal DCs, and dermal DCs. As there is an ongoing discussion about the function of different DC subpopulations in the skin, we also analyzed these cells in WT and Cytip KO mice. For this, LN cells were stained for CD11c, Langerin, and CD103. Like the LC number in the epidermis, there was no difference detectable regarding the DC populations in skin draining LNs (Fig. 1C).

Culture of bone marrow-derived DCs (BMDCs) from either Cytip KO or WT animals resulted in the same yield of DCs (data not shown).

### Cytip-deficient DCs show an increased T-cell activation capacity

**Figure 2:**
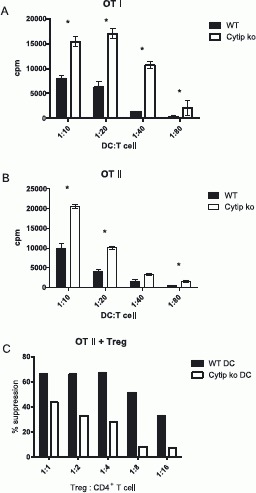
Cytip-deficient DCs induce higher proliferation in both OT-I and OT-II T cells. BMDCs cultured from either WT or Cytip KO bone marrow were loaded at day 6 of culture with 20 or 500 μg/mL whole OVA and CpG for 18 h and cocultured with either (A) MACS-sorted OT-II (CD4^+^) or (B) OT-I (CD8^+^) T cells, respectively, at varying ratios. Proliferation was measured after 4 days of culture by ^3^H-thymidine incorporation. Data are shown as mean + SD of triplicates and are representative of three independent experiments. *p < 0.05 versus WT, Student's t-test.

To investigate the functional role of Cytip in mouse DCs, we first performed a mixed leukocyte reaction (MLR) with BMDCs generated from WT and Cytip KO mice as antigen-presenting cells (APCs). BMDCs were cultured for 6 days, activated with cytosine guanine dinucleotide (CpG) and loaded with whole ovalbumin (OVA) protein at the same time for 18 h and cocultured either with OT-I (CD8^+^) or OT-II (CD4^+^) T-cells (where OT is OVA specific TCR transgenic mice) in different ratios (Fig. 2A and B). Both OT-I and OT-II T cells displayed a higher proliferation when Cytip KO DCs were used as APCs independently of the ratio between T cells and DCs. Additionally, we added regulatory T cells from OT-II cells to cocultures of OT II and BMDCs (Fig. 2). Proliferation of CD4^+^ T cells was suppressed to 70% when WT BMDCs were used for stimulation. In comparison, regulatory T cells suppressed CD4^+^ T-cell proliferation just up to 40%. So taken together, BMDCs from Cytip-deficient mice stimulate CD4^+^ and CD8^+^ T cells much better than their WT counterparts and abrogate the suppressive capacity of regulatory T cells to a certain degree.

Based on the results, we wanted to investigate why Cytip-deficient DCs show this improved potential to activate T cells. For this reason, BMDCs generated from WT and Cytip-deficient mice were cultured for 6 days. After 18 h of CpG-mediated activation, DCs were stained for molecules (PDL-1/2, ICOSL, GITRL, CD80/86, CD40, and MHC class II), which might influence the activation of T cells by DCs. No difference was detectable between WT and Cytip KO DCs regarding the expression of costimulatory molecules (data not shown).

### Cytip-deficient DCs strengthen CHS reactions

**Figure 3:**
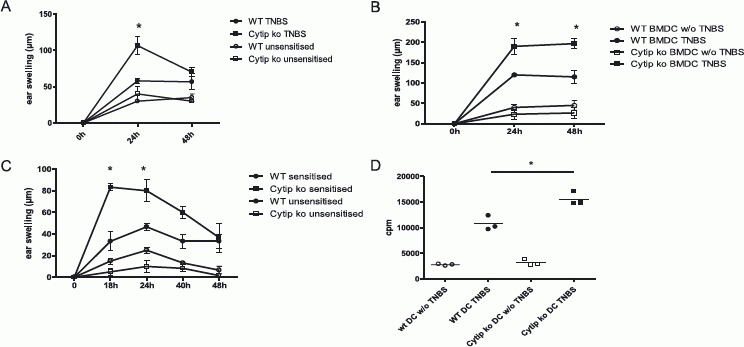
Cytip KO DCs increase the sensitization and elicitation phases of contact hypersensitivity. (A) WT and Cytip-deficient mice were sensitized with 1% TNCB on the shaved abdomen. Five days later, mice were challenged with TNCB on both sides of the ear and ear swelling was measured at 24 and 48 h. As control, unsensitized mice were challenged with TNCB. (B) BMDCs from WT or Cytip KO mice were activated, loaded with TNBS and injected intradermally into WT recipients. Five days later, mice were challenged with TNCB and ear swelling was measured. As a control DCs without TNBS treatment were injected. (C) WT mice were sensitized on the shaved abdomen with 1% TNCB. On day 5, spleen cells of these mice were prepared and injected intravenously (i.v.) into WT or Cytip-deficient mice. One hour later, recipient mice were painted with TNCB. Ear swelling was determined. As a control, WT and Cytip-deficient mice were injected with spleen cells from untreated animals. (D) WT mice were sensitized on the ear with TNCB and 5 days later, cell suspensions from draining LNs were prepared. LN cells were restimulated with TNBS-treated BMDCs or as controls, untreated BMDCs from WT or Cytip KO mice. Proliferation was measured after 4 days of culture by ^3^H-thymidine incorporation. Data are shown as mean ± SD of *n* = 3 mice per group and are representative of three independent experiments. *p < 0.05 versus WT, Student's t-test.

Due to the increased proliferation of OT-I and OT-II T cells when Cytip KO DCs were used as APCs (shown in Fig. 2), we wanted to investigate the function of Cytip in DCs in vivo. For this we used the model of CHS to define the role of DCs in both the priming and effector phase of immune responses. WT and Cytip-deficient mice were painted on the shaved abdomen with 2,4,6-trinitrochlorobenzene (TNCB), 5 days later, mice were challenged with TNCB on the ear skin and ear-swelling reaction was measured up to 48 h. As control, unsensitized mice were challenged with TNCB (Fig. 3A). In this setting, Cytip-deficient mice showed an increased ear-swelling reaction after 24 h compared with WT mice. After 48 h ear swelling in Cytip-deficient mice decreased to WT level. To define DC function in the priming phase, BMDCs were activated with CpG and loaded with 2,4,6-trinitrobenzenesulphonic acid (TNBS). As a control, BMDCs without TNBS treatment were used. WT or Cytip KO BMDCs were injected intradermally into WT recipients and 5 days later, mice were challenged with TNCB on the ear skin and ear-swelling reaction was followed over 48 h. Cytip-deficient DCs induced a higher ear-swelling reaction than their WT counterparts when they were used for priming CHS (Fig. 3B). This is in accordance with the in vitro assays shown in Fig. 2. To examine whether Cytip plays a role in DCs during effector phase, WT mice were sensitized with TNCB on the shaved abdomen 5 days before preparing spleen cells from these mice. Cells were injected intravenously (i.v.) into WT or Cytip KO recipients and challenged 1 h later with TNCB. Ear-swelling reaction was measured up to 48 h (Fig. 3C). In this setting, DCs from WT or Cytip-deficient recipient mice restimulated TNCB primed T cells from sensitized WT mice. Also in this experiment, ear-swelling reactions were increased, when DCs were Cytip deficient. Transfer of spleen cells without former sensitization did not induce any ear-swelling reaction in recipient mice after TNCB treatment. Challenging Cytip-deficient mice, which were primed with WT TNBS- DCs, resulted in a slightly decreased ear-swelling reaction compared with their WT counterparts (Supporting Information Fig. 1A). Also the transfer of spleen cells from TNCB sensitized Cytip-deficient mice induced a lower ear-swelling reaction after TNCB challenge in WT mice compared with spleen cells from sensitized WT mice, although these differences were not statistically significant (Supporting Information Fig. 1B).

To underline these results, cell suspensions from skin draining LNs from TNCB-treated WT mice were prepared 3 days after treatment. Cells were restimulated with CpG-activated and TNBS-loaded BMDCs from either WT or Cytip-deficient mice in vitro for 3 days, as control unloaded BMDCs were used (Fig. 3D). Proliferation could be detected in both restimulation approaches, either with WT or Cytip KO DCs. Unloaded DCs failed to induce any proliferation. Restimulation with Cytip KO DCs showed a significant higher proliferation of sensitized LN cells comparing to WT DCs.

### Migration of Cytip KO DCs is not altered following local TNCB treatment

**Figure 4:**
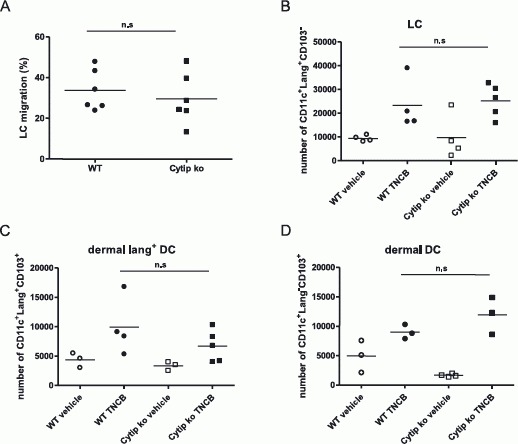
DC migration after TNCB treatment is not altered in Cytip KO mice. Epidermal sheets from WT and Cytip KO mice were stained for MHC class II and LCs were counted. (A) Epidermal sheets were prepared 2 days after TNCB painting and stained for MHC class II, and LC migration was calculated by comparing LC numbers in TNCB- and vehicle-treated epidermis within the same animal. (B) LN cells were isolated 3 days after TNCB or vehicle treatment and single cell suspensions were stained for CD11c, Langerin, and CD103 to assess the migration of different DC subpopulations ((B) LC, (C) Lang^+^dermal DCs, and (D) dermal DCs) into skin draining LNs. Total numbers of DCs per LN in vehicle- or TNCB-treated draining LNs of WT and Cytip KO mice are shown. Data are shown as mean of 3–5 mice per group and are representative of three independent experiments.

As it has been published that contact sensitizers induce the migration of DCs [12], we investigated the migration of different DC populations in the skin following TNCB treatment to exclude that different migration of WT or Cytip-deficient DCs might influence the outcome of CHS. To follow the migration of LCs, we treated one ear of WT and Cytip KO mice with TNCB and one with vehicle once and prepared epidermal sheets after 3 days. Sheets were stained for MHC class II and LCs were counted. Emigration was calculated by comparing TNCB- and vehicle-treated ear skin within the same animal. As shown in Fig. 4A, there was no difference in LC emigration from the epidermis in WT or Cytip KO mice after TNCB treatment.

We also prepared LN cells suspension from skin draining LNs 3 days after TNCB or vehicle painting and investigated the total cell numbers of DC subpopulations (Fig. 4B-D). As described in Fig. 1A for untreated animals, no difference could be found between Cytip KO and WT mice in draining LNs of vehicle or TNCB-treated skin. All skin DC subpopulations migrate into the LNs after TNCB treatment to the same extent in WT or Cytip-deficient mice.

Additionally, we examined the expression of ICAM-1 and LFA-1 (CD18/CD11a) on WT and Cytip-deficient DCs as these molecules are involved in DC migration [13] and as it was published that Cytip might regulate the function of LFA-1 [14]. But we could not find any difference either in unstimulated or CpG-activated DCs (data not shown).

### CpG stimulation decreases Cytip mRNA and increases IL-12 expression in Cytip-deficient BMDCs

**Figure 5:**
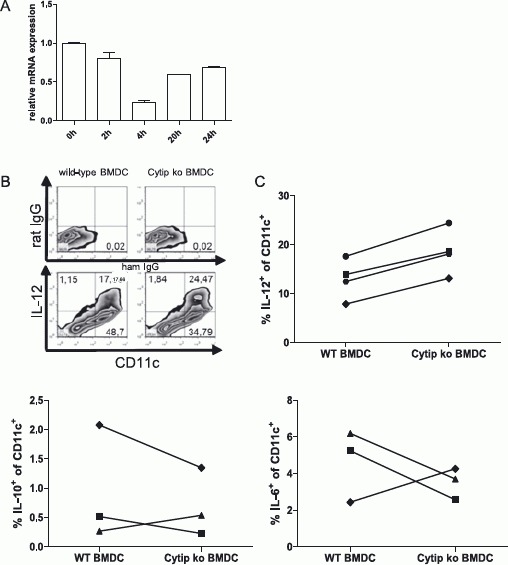
CpG stimulation down regulates Cytip mRNA expression and leads to higher IL-12 expression in Cytip-deficient BMDCs. DCs were generated from Cytip KO or WT bone marrow and cultured for 6 days. (A) For Cytip mRNA detection, WT BMDCs were activated with CpG for the indicated time and Cytip expression was determined with qPCR. Data represent relative mRNA expression and are representative for three independent experiments. (B) For flow cyto-metric analysis of IL-12, IL-6, and IL-10 production, cells were stimulated for 18 h with CpG and Golgi block was added for the last 4 h. Cytokine production by DCs was determined by staining with antibodies against CD11c and intracellular IL-12, IL-6, and IL-10. As a control unstimulated DCs were stained with isotype antibodies. Shown is (B) one representative flow cytometry plot and (C) a summary of four independent measurements, each symbol indicating a single experiment.

To investigate the kinetics of Cytip mRNA expression, we stimulated WT BMDCs with CpG and detected Cytip mRNA by quantitative polymerase chain reaction (qPCR) at different time points (Fig. 5A). Cytip mRNA was downregulated after 4 h of CpG stimulation and after 24 h Cytip mRNA was expressed almost to the same extent as in unstimulated cells but never reached the level of unstimulated BMDCs again.

We also analyzed cytokine production of CpG-activated BMDCs from both mouse strains. Golgi stop was added for the last 4 h of stimulation. Cells were stained for CD11c to identify DCs, and fixed and stained for intracellular IL-12, IL-6, and IL-10 (Fig. 5B and C). WT as well as Cytip KO BMDCs produce IL-12 after CpG stimulation, but in all experiments Cytip-deficient BMDCs show an increased cytokine production compared with that of their WT counterparts. IL-6 and IL-10 expression were not affected by Cytip deficiency (Fig. 5B and C), both WT and Cytip KO produce these cytokines to the same extent after CpG activation.

## Discussion

Mature DCs are the major cell type in directing and regulating specific immune responses. Cytip is one of the molecules induced during maturation of human DCs and it was shown to be involved in the regulation of immune responses. Cytip is also induced in T cells during priming/activation phase of antigen-specific immune responses [15]. Therefore, the role of Cytip in the regulation of immune responses could be mediated both by T cells and/or DCs. Here, we aim at characterizing the role of Cytip in murine DCs by functional comparison of DCs from Cytip KO mice and WT mice. In accordance with the characterization of the two Cytip-deficient mouse strains available [7, 8], both showing that Cytip deficiency does not affect the development of the immune system, we did not find major differences in the numbers of DCs in skin draining LNs. To further define the role of Cytip in DCs, we compared the expression of molecules induced during maturation of WT and Cytip KO mice upon CpG-induced maturation. We found that Cytip deficiency does not alter the expression of surface or costimulatory molecules on DCs. In WT and Cytip KO BMDCs expression was equally regulated after CpG activation.

Human DCs silenced for Cytip with small interfering RNA (siRNA) show a reduced stimulatory capacity for antigen-specific CD8^+^ T cells in vitro when only a part of the DCs are loaded with antigen. This was interpreted by the authors as an impaired screening for MHC/peptide-TCR matches [5]. Similarly, Cytip-deficient mice have a weaker immune response in a virus-induced tumor model [7], but it is not clear whether this is due to the Cytip deficiency in T cells or in DCs or both. To define the role of Cytip in mouse DCs, we loaded Cytip-deficient or WT BMDCs with whole OVA protein and used them as APCs. We observed a higher proliferation of OT-I (CD8^+^ T cells) and OT-II (CD4^+^ T cells) when Cytip-deficient DCs were used. Also suppression by regulatory T cells was reduced when antigen was presented by Cytip-deficient BMDCs. The difference seen in this setting is clearly DC linked because the same OT-I/OT-II cells were used as responders with WT or Cytip KO BMDCs as APCs. Differences seen between results from Hofer et al. [5] and our results might be because of different species (human/mouse) or different protocols regarding antigen loading and T-cell stimulation. Also different methods to impede Cytip expression in DCs, siRNA in human cells, or complete KO in murine cells, might play a role in receiving different results. Balkow et al. [14] showed recently, that DCs silenced for Cytip induced less proliferation of OT-II T cells than DCs treated with control siRNA. These different results could also be due to different protocols used to modulate Cytip expression in DCs.

CHS reaction induced by TNCB application onto the skin is a T-cell-mediated inflammatory disease. During sensitization phase, DCs loaded with hapten migrate to skin draining LNs and induce priming of hapten-specific T cells [12]. We used a CHS model to verify a role of Cytip in DCs in vivo. First, we sensitized WT and Cytip-deficient mice with TNCB and challenged them with TNCB 5 days later. Both showed ear swelling reaction after 24 h but Cytip-deficient mice had increased inflammation upon TNCB challenge. To investigate the role of Cytip in DCs during CHS in more detail, we injected WT or Cytip-deficient TNBS-loaded BMDCs into the back skin of WT recipients. We found a more pronounced immune response in mice that were injected with Cytip-deficient TNBS-loaded BMDCs as compared with WT TNBS-loaded DCs indicating a stronger sensitization with Cytip-deficient DCs. This enhanced sensitization is not due to different migration properties of WT and Cytip-deficient BMDCs since Watford et al. demonstrated that both WT and Cytip-deficient BMDCs migrate equally after injection [8]. During challenge phase, hapten-specific T cells migrate into the skin and induce the inflammatory reaction [16]. To investigate whether Cytip deficiency in DCs also plays a role during effector phase, we injected hapten-primed WT T cells into WT or Cytip-deficient mice and challenged them with TNCB. Again we observed an increased ear swelling, when Cytip KO DCs present the hapten. Since the responder T cells were WT in both settings, the differences seen can again be attributed to the Cytip deficiency in DCs. In accordance with the proliferation assays with OT-I and OT-II T cells, restimulation of TNCB sensitized WT LN cells with TNBS-loaded BMDCs from WT or Cytip-deficient mice, we also observed an increased proliferation of LN cells when the hapten was presented by Cytip-deficient BMDCs. Therefore, in this in vivo model again the differences seen are solely mediated by DCs as responder T cells are WT in both settings. If T cells are also Cytip deficient, ear-swelling reaction seems to be slightly decreased or the difference seen with Cytip-deficient DCs alone disappear. After transfer of primed Cytip-deficient spleen cells into WT recipients or after sensitization of Cytip-deficient mice with WT DCs, ear-swelling reaction decreased after TNCB challenge. So there might be a defect in Cytip-deficient T cells, which compensates the increased stimulatory capacity of Cytip-deficient DCs. This might also be the reason for the reduced immune response in the virus-induced tumor model [7].

The increased immune response might be due to the longer contact time between hapten-bearing DCs and specific T cells as described for human DCs [5] thus increasing the activation and proliferation of T cells. It is yet unclear which DC subpopulation induces or suppresses CHS as there are different ablation models for skin DCs and different CHS protocols available [9–11]. These publications show that LCs seem to suppress or to enhance or are not necessarily essential to induce CHS respectively. It was also shown that Cytip might affect the migration of lymphocytes. Therefore, we investigated the different DC populations found in draining LNs after TNCB application in Cytip-deficient mice compared with their WT littermates. We could not find any difference in migration of DC subpopulations into the skin draining LNs and also skin draining LN cell numbers show no difference between WT and Cytip KO mice. In addition, several studies have shown that IL-12 production by DCs plays a role during sensitization phase of CHS [17, 18]. We showed in this study that DCs stimulated with CpG downregulate the mRNA expression for Cytip while starting to produce IL-12. Additionally, Cytip-deficient DCs produced an increased amount of IL-12 after activation with CpG. This could also contribute to their enforced capacity to stimulate T cells and potentiate CHS reaction. The molecular mechanisms how Cytip might regulate cytokine production and T-cell activation remain to be elucidated.

## Materials and methods

### Mice

Inbred C57Bl/6 mice, OT-I and OT-II mice, expressing the trans-genic Vα2Vβ5.1/5.2 TCR specific for K^b^ + OVA _257–264_ and I-A^b^ + OVA _323–339_, respectively, were purchased from Charles River Laboratories (Sulzfeld, Germany) and used at the age of 8–12 weeks. Cytip-deficient mice [7] were kindly provided by Antonio Rosato (Department of Oncology and Surgical Sciences, University of Padova, Italy).

All animals were bred at the animal facility of Innsbruck Medical University. All animal experiments were performed in accordance with governmental guidelines.

### Media and reagents

Complete culture medium was provided by Roswell Park Memorial Institute-1640 (PAA Laboratories, Linz, Austria) supplemented with 10% heat-inactivated fetal calf serum (Lonza-BioWhittaker, Verviers, Belgium), 2 mM L-glutamine (Sigma-Aldrich, St. Louis, MO), 50 μg/mL gentamicin (PAA, Linz, Austria), and 50 μM β-mercaptoethanol (Sigma-Aldrich). For generation of BMDCs, GM-CSF (200 U/mL) containing supernatant (transfected plas-macytoma cell line X38-Ag8, A. Lanzavecchia, Bellinzona, CH) was added to the medium.

### Generation and stimulation of BMDCs

Immature DCs from Cytip-deficient or from WT mice (C57BL/6) were generated from bone marrow according to standard protocols [19]. In brief, DCs were differentiated with GM-CSF for six days with medium replacement on days 2 and 4. On day 6, immature DCs were partially activated with 1 μg/mL CpG 1826 (TIB, Molbiol, Hamburg, Germany) as indicated.

Loading of DCs with 1 mM 2, 4, TNBS (Sigma-Aldrich) was done after 1 h CpG stimulation for 7 min at 37°C. Cells were washed once with culture medium and twice with PBS.

For T-cell proliferation assays BMDCs were incubated with 20–500 μg/mL whole OVA protein (Sigma-Aldrich) for 18 h.

### FACS analysis

Cells were stained for 20 min with 0, 5–1 μg/mL of the indicated antibodies. All staining and washing steps were done at 4°C. Intracellular staining for cytokines and Langerin were performed after permeabilization of cells with Cytofix/Cytoperm kit (BD Biosciences, San Diego, CA), according to manufacturer's instruction. For detection of intracellular cytokines, Golgi stop (BD Biosciences) was added for the last 4 h of stimulation.

The following antibodies were purchased from BD Biosciences: CD103 biotin (clone M209), Streptavidin-allophycocyanin, IL-12-PE (clone C15.6), IL-10-PE (clone JES5-16E3), PDL-1-PE (clone MIH5), PDL-2-PE (clone TY25), LFA-1-PE (clone C71/16), B220-FITC (clone RA3-6B2), CD86-PE (clone GL1), CD40-PE (clone3/23), and ICAM-1-PE (clone 3E2), ICOSL-PE (clone HK5.3), CD11c-PECy5 (clone N418), CD8-PE (clone 53-6.7), IL-6 (clone MP5-20F3), and GITRL-PE (clone YGL386) were obtained from ebioscience (San Diego, CA), antibody against Langerin (clone 929F3-FITC) was purchased from Dendritics (Lyon, France). CD4-PE antibody (clone GK1.5) was purchased for BioLegend (San Diego, CA).

For FACS analysis of DCs in skin draining LNs, single cell suspension was prepared from auricular LNs 2 days after painting with 20 μL 1% TNCB (Sigma-Aldrich) on both sides of the ear or vehicle treatment as previously described [12].

BMDC were stimulated with 1 μg/mL CpG for 18 h and stained either for costimulatory molecules or cytokine production.

All samples were analyzed on fluorescence-activated cell sorter (FACS) Calibur using CellQuest software (BD Biosciences) and analyzed by FlowJo (Tree Star).

### mRNA detection

Ribonucleic acid (RNA) was isolated using TRIzol (Invitrogen) and complementary deoxyribonucleic acid (cDNA) was synthesized with Superscript II ribonuclease (RNase) H-reverse transcrip-tase (Life Technologies, Vienna, Austria) from total RNA. Realtime PCR was performed using the following oligonucleotides: TATA-binding protein forward ACT TCG TGC AAG AAA TGC TGC TGA A, TATA-binding protein reverse TGT CCG TGG CTC TCT TAT TCT CA, TaqMan primer TCC CAA GCG ATT TGC TGC AGT CAT C, Cytip forward GGA AGA CAG CCC CGC TCA C, Cytip reverse GCT CAC ACC ATT GAC ATT TGC AAA G, TaqMan primer TGC TGG CCT GCA AGT TGG TGA. Real-time PCR analyses to quantify the expression of mRNAs were performed in triplicates on an ABI PRISM 7700 sequence detector (Applied Biosystems, Vienna, Austria) using the Brilliant Quantitative PCR Core Reagent Kit from Stratagene (Heidelberg, Germany). After normalization of the data according to the expression of TATA bp mRNA, the relative expression levels of Cytip mRNAs were calculated.

### Preparation of T-cell populations

Conventional CD4^+^CD25^−^ T cells from OT-II mice were prepared from spleen cells using magnetic-activated cell sorting beads (MACS-beads) (Miltenyi Biotec, Bergisch Gladbach, Germany) according to the manufacturers' instructions. For this, cells were incubated with a biotinylated anti-CD4 mAb (clone H129.19, BD Biosciences) and after two washing steps, with streptavidine-coupled microbeads (Miltenyi Biotec). CD8^+^ T cells from OT-I mice were prepared with anti-CD8 mAb conjugated microbeads. To prepare Treg cells, spleen cells were incubated with a biotinylated anti CD25 antibody (clone 7D4, BD Biosciences) and after two washing steps, with streptavidine-coupled microbeads (Miltenyi Biotec). After MACS sorting, positive selected cells were further enriched by depleting CD8^+^ T cells, B cells, and macrophages with Dynabeads (Invitrogen). All sortings on columns were done twice.

### T-cell stimulation and proliferation assays

A total of 2 × 10^5^ CD4^+^CD25^−^ T cells from OT-II mice were incubated with graded numbers of OVA-loaded DCs in a total volume of 200 μL in 96 flat bottom plates for 4 days. For suppression assays 2 × 10^5^ regulatory T cells from OT II mice were added to the cocultures. A total of 2 × 10^5^ CD8^+^ T cells prepared from OT-I mice were also incubated with graded numbers of OVA loaded DCs for 3 days in 200 μL. In all cultures, ^3^H-thymidine was added for the last 18 h and uptake was assessed by β scintillation counting.

### Induction of CHS reaction

To investigate the role of Cytip in DCs during sensitization or elicitation phase of CHS, CHS was induced using two different models.

For sensitization, TNBS-loaded or -unloaded DCs (3 × 10^5^ in 20 μL PBS) were injected intradermally into two sides of the shaved abdomen. Five days later, mice were challenged by painting 20 μL 1% TNCB (Sigma-Aldrich) in acetone olive oil (4:1) on both sides of the ear. Ear-swelling reaction was measured up to 48 h.

For elicitation mice were sensitized on the shaved abdomen with 200 μL 1% TNCB in acetone olive oil (4:1). Five days later, spleen cells from these or from not sensitized mice were transferred i.v. (2 × 10^7^ in 200 μL PBS) into untreated recipients. These recipient mice were treated with 20 μL of 1% TNCB on both sides of one ear 1 h after i.v injection. Ear swelling was measured up to 48 h.

Epidermal sheets from mice treated with 20 μL 1% TNCB or vehicle on both sides of the ear were prepared 2 days after painting as described previously [20] and stained for MHC class II (clone M5.114.15.2) and an Alexa488-labeled secondary goat anti rat antibody (Molecular Probes, Eugene, OR). MHC class II-positive cells were counted per region of interest, percent migration was calculated by comparing numbers of MHC class II-positive cells from TNCB- and vehicle-treated skin within the same animal. Four light power fields were counted per mouse.

For LN restimulation, mice were painted on both sides of the ear with 1%TNCB. Five days later, draining LNs were harvested and a single-cell suspension was prepared. LN cells were restimu-lated with TNBS-treated BMDCs for 3 days and ^3^H-thymidine was added for the last 18 h and uptake was assessed by β scintillation counting.

### Statistical analysis

Data are expressed as mean ± SD. Student's *t*-test was used to compare mean values between two experimental groups where appropriate. Values of *p* < 0.05 were considered significant.

## Abbreviations

BMDC: bone marrow-derived dendritic cell
CHS: contact hypersensitivity
Cytip: Cytohesin Interacting protein
LN: lymph node
TNBS: 2,4,6-trinitrobenzenesulphonic acid
TNCB: 2,4,6-trinitrochlorobenzene
